# Controlled and Accelerated Hydrolysis of Polylactide (PLA) through Pentaerythritol Phosphites with Acid Scavengers

**DOI:** 10.3390/polym14194237

**Published:** 2022-10-10

**Authors:** Matthias Polidar, Elke Metzsch-Zilligen, Rudolf Pfaendner

**Affiliations:** Fraunhofer Institute for Structural Durability and System Reliability LBF, Division Plastics, 64289 Darmstadt, Germany

**Keywords:** hydrolysis, poly(lactic acid), polylactide, PLA, controlled degradation, accelerated degradation, phosphite

## Abstract

This study provides insight into the accelerated hydrolysis of polyester PLA through the addition of phosphites based on pentaerythritol. To control hydrolysis and ensure processing stability, different types of phosphites and combinations of phosphites with acid scavengers were studied. Therefore, commercially available PLA was compounded with selected additives on a twin-screw extruder, and hydrolysis experiments were performed at 23 °C, 35 °C and 58 °C in deionized water. Hydrolysis of PLA was evaluated by the melt volume rate (MVR) and size-exclusion chromatography (SEC). For example, after 4 days of water storage at 58 °C, the number average molecular weight of the PLA comparison sample was reduced by 31.3%, whereas PLA compounded with 0.8% phosphite P1 had a 57.7% lower molecular weight. The results are in good agreement with the expected and tested stability against hydrolysis of the investigated phosphite structures. ^31^P-NMR spectroscopy was utilized to elucidate the hydrolysis of phosphites in the presence of lactic acid. With the addition of phosphites based on pentaerythritol, the hydrolysis rate can be enhanced, and faster biodegradation behavior of biodegradable polyesters is expected. Accelerated biodegradation is beneficial for reducing the residence time of polymers in composting facilities or during home composting and as litter or microplastic residues.

## 1. Introduction

Biobased and biodegradable polymers are considered vital components of a rising circular economy [1,2], and there is an urgent need to reduce waste that is not appropriately recycled but discarded in the environment through littering or in landfill sites [3,4]. Biodegradability offers a suitable end-of-life option for certain products and, at the same time, reduces the residence time of litter and plastic debris [5]. Provided that a polymer is considered theoretically biodegradable, which means that the polymer chains are cleaved and metabolized by microorganisms in a reasonable timescale, this process often needs to be accelerated and ideally controlled.

Aliphatic polyesters such as polylactide (PLA) are promising candidates for the substitution of fossil-based and non-biodegradable polymers, such as polystyrene and poly(ethylene terephthalate), due to their similar properties, good processability and increasing production capacity, which consequently is expected to lead to competitive costs [6,7,8]. Commercial PLA is biodegradable, provided that high temperatures of around 60 °C, humidity and microorganisms are available, as in industrial composting facilities [9]. Apart from those conditions, the biodegradation of PLA is rather slow and comparable to its non-biodegradable counterparts [9]. For example, it was reported that PLA films only slightly disintegrated over 11 months in Mediterranean soil [10]. Moreover, the time to be fully composted during industrial composting often outlasts one composting cycle and, therefore, must also be accelerated [11].

The biodegradation of PLA can be described in two stages. First, there is a preliminary abiotic step to cleave the macromolecular chains into monomers and oligomers. For polyesters such as PLA, this is usually the hydrolysis of ester bonds initiated by the absorption of water. Specific microorganisms can mineralize these so-formed lower-weight molecules in a second step, which can be described as proper biodegradation [8,12].

By the end of the last century, extensive research had been conducted in terms of the hydrolytic degradation behavior of PLA and its blends, but mainly in phosphate buffer solution at 37 °C to simulate a human body-like environment for medical devices or drug delivery [13,14,15,16,17,18]. More recently, with priority given to environmental concerns and the more attractive price of PLA facilitating its use for everyday products, the focus has shifted towards increased research on the hydrolysis and degradation behavior of PLA under environmental and composting conditions [12,19,20,21,22,23,24].

The hydrolysis of PLA is well known to depend on pH and to proceed autocatalytically with the formation of acidic degradation products [25,26]. Generally speaking, PLA hydrolysis is accelerated in acidic or alkaline conditions [27,28,29,30]; however, the influence of the pH of the testing media on the molecular weight of PLA is restricted when bulky specimens are tested [31,32,33]. Hydrolysis can proceed via random- or end-chain scission [29]. Ester groups near the terminal groups of oligomeric PLA were found to be more prone to hydrolysis [34]. Nevertheless, random-chain scission plays a decisive role, as molecular weight reduction is observed quickly during PLA hydrolysis, taking into account the large proportion of non-terminal ester groups in high molecular weight PLA [35]. On a macroscopic scale, a bulky specimen can hydrolyze faster internally, while the surface still may be intact, which is then termed “bulk erosion” and occurs when water diffusion is higher than the hydrolysis rate, whereas “surface erosion” describes a faster reduction in volume caused by hydrolysis and subsequent mass loss at the surface of a specimen, while the molecular weight inside remains constant [27,36,37]. Theoretically, all hydrolyzable polymers can erode in both patterns, depending on water diffusivity, the degradation rate and the dimensions of the specimen [37]. For PLA in alkaline media, surface erosion was reported, whereas in neutral or acidic media, bulk erosion for PLA was found to be predominant [32,37]. Autocatalytic bulk erosion was proposed to describe the heterogeneous bulk erosion of PLA specimens enhanced by the autocatalytic effect of acidic degradation products accumulating inside [36].

Besides pH, temperature is a major factor when investigating PLA hydrolysis. Lyu et al. [33] examined the hydrolysis of PLA over a temperature range from 37 to 90 °C at pH 7.4 in phosphate buffer solution (PBS). At 90 °C, 1 mm thick samples were completely dissolved after 2 days, whereas at 37 °C, samples were tested for up to 500 days. In several more studies, the temperature-dependent hydrolysis of PLA was investigated [22,32,38]. In summary, it can be stated that there is a strong dependency of the hydrolysis rate on the testing temperature. In particular, hydrolysis is restricted below glass transition (55–60 °C), which is explained by the restriction of movement by the effective catalytic chain ends below $T_{g}$ [33,39].

Some approaches have been described to accelerate the hydrolysis rate of biodegradable polyester PLA. Firstly, increasing the concentration of carboxylic end groups available to initiate acid-catalyzed hydrolysis has been evaluated by blending it with designed polymers characterized by a large number of acidic end groups [40,41,42,43], adding fumaric acid [44] or lauric acid [45] or using a higher oligomer concentration [46,47]. For example, adding 4.5% lauric acid to PLLA accelerated the degradation at 37 °C by a factor of 5–10, and about 3% of the remaining ester groups were cleaved daily [45]. The influence of D-units of lactic acid on hydrolysis was also investigated [17,18,48]. Surface treatment such as vapor-phase grafting with acrylic acid led to faster formation of water-soluble degradation products [49]. Blending PLA with more hydrophilic polymers such as poly(vinyl alcohol) enhances the hydrolysis rate [50]. Furthermore, copolymers of lactide were tested, e.g., with glycolide [18,46,51], dextrans [52] and salicylic methyl glycolide [53]. For example, amorphous films from copolymers of glycolide and lactic acid showed a hydrolytic reduction in molecular weight at 37 °C by half in about 10 weeks, whereas the PLLA molecular weight was nearly unchanged [18]. The additive solutions published so far are based on citrate esters or caffeine. Citrate esters added as plasticizers led to higher weight loss during hydrolytic degradation [54], but other authors observed that acetyl tributyl citrate (ATC) reduced the hydrolysis of PLA [55]. Using caffeine as a base at low concentration led to faster degradation at first, possibly by base catalysis, and subsequently neutralized new carboxyl groups formed, thereby suppressing autocatalysis and increasing hydrolytic stability [16].

Triarylphosphites act as processing aids by acting as chain extenders for PLA [56,57,58,59]. Recently, the phosphite tris(nonylphenyl)phosphite (TNPP), but not tris(2,4-di-tert-butylphenyl)phosphite (TDBP), possibly due to higher hydrolytic stability, has been found to accelerate the hydrolysis of PLA [59]. TNPP has negative biological effects and is suspected to act as an endocrine disruptor by releasing nonylphenol (NP) as an impurity and hydrolytic degradation product [60]. Therefore, TNPP is listed as a substance of very high concern [61] and is expected to be phased out for sustainable polymer applications. Some phosphites based on pentaerythritol can overcome these shortcomings, and research data on their influence on processing stability and hydrolytic degradation of PLA are lacking.

The hydrolysis pathway of pentaerythritol-based diphosphites was described by Ortuoste et al. [62]. In the first step, the peripheral P-O-R bonds of the spiro compound are hydrolyzed. After subsequent hydrolysis steps, finally, phosphorous acid, pentaerythritol and alcoholic compounds are formed. The hydrolytic stability [63] and the stabilizing performance [64] of pentaerythritol diphosphites depend on the structure of the organic rest R. The hydrolysis of pentaerythritol diphosphites proceeds autocatalytically, and chemical compounds formed with the ability to react with acidic species, named acid scavengers, inhibit autocatalytic hydrolysis [62].

The aim of this study is to identify suitable additives for the controlled accelerated hydrolytic degradation of PLA while maintaining processing stability during compounding and manufacturing. As a potential class of phosphites, commercially available pentaerythritol derivatives were selected and combined with selected acid scavengers to achieve temperature and timewise control of the degradation.

## 2. Materials and Methods

### 2.1. Materials

PLA is characterized by its content of L,L-lactide, D,L-lactide and D,D-lactide. The PLA grade used in this study was Luminy L175, a commercially available and fully biobased PLLA with at least 99% L-isomer content (supplier information), supplied by TotalEnergies Corbion, Gorinchem, Netherlands. The high content of L-lactic acid distinguishes PLLA from PDLA: the latter contains higher content of D-lactic acid, which leads to different material properties, e.g., limited degree of crystallinity. The phosphites investigated were distearyl pentaerythritol diphosphite (Weston 618F, supplied by SI Group, The Woodlands (TX), USA, referred as P1), bis(2,4-di-tert-butylphenyl) pentaerythritol diphosphite (Songnox 6260 PW, supplied by Songwon, Ulsan, Korea, P2) and bis(2,4-dicumylphenyl) pentaerythritol diphosphite (Doverphos S-9228, supplied by Dover Chemical Corporation, Dover (OH), USA, P3). Pentaerythritol diphosphites were used, because pentaerythritol is a potentially biobased substance, and phosphites based on it are widely commercially available. Moreover, as a platform chemical, pentaerythritol offers many possibilities for modified chemical compounds. As acid scavengers, calcium stearate (Caesit AV, supplied by Baerlocher, Unterschleißheim, Germany, S1) and a fine powder magnesium/aluminum-hydrotalcite (Hycite 713, supplied by Clariant, Muttenz, Switzerland, S2) were used.

### 2.2. Extrusion

Compounding was performed on a co-rotating parallel twin-screw extruder Process 11 (Thermo Fisher Scientific, Karlsruhe, Germany) with a Length-to-Diameter ratio of 40. Prior to the extrusion process, pellets of L175 were cooled in liquid nitrogen and milled on an impact plate mill (PPL18, Pallmann Maschinenfabrik GmbH & Co. KG, Zweibrücken, Germany) with a gap width of 3 mm. Before the extrusion process, milled PLA was dried by vacuum drying for 1 h at room temperature and 16 h at 80 °C. The phosphite Weston 618F was delivered as flakes and mortared and weighed in an inert glove box to ensure no hydrolytic degradation prior to processing. The other phosphites were delivered as powders. Hydrotalcite was dried in a vacuum oven (5 mbar) at 150 °C for 16 h prior to the extrusion process. Polymer and additives were mixed in a bag and added to the volumetric dosage unit, which was set to a mass throughput of 800 g per hour. The screw speed of the extruder was set to 200 rpm, and the temperature profile was set to increase from 180 °C in the feeding zone to 200 °C in the mixing zones and the die. Vacuum degassing, a water bath and a pelletizer were used. The extruded pellets were dried under reduced pressure at room temperature for at least 24 h and stored in vacuum bags prior to hydrolysis experiments.

### 2.3. Hydrolysis Test

For hydrolysis tests, 10 g of pellets were added to a glass jar, and deionized water was added to attain a weight ratio of pellets to water of 1:20. The glass was closed with a lid and placed in a convection oven (Binder GmbH, Tuttlingen, Germany), which was set to the respective testing temperatures of 35 and 58 °C. Tests at 23 °C were conducted in a standardized climate. Temperatures were selected with regard to different degradation environments: industrial composting conditions (58 °C, which is also around the glass transition temperature of PLA), home composting (35 °C) and ambient degradation (23 °C). For each removal time, duplicates were prepared. The water was decanted, and the granulate was sieved and subsequently dabbed with a lint-free disposable paper and dried in a vacuum oven at room temperature for at least 2 h and at 80 °C for 16 h. At the beginning and at every removal time, pH was measured with a pH meter (FiveEasy Plus™ pH FP20, Mettler-Toledo GmbH, Gießen, Germany) equipped with a glass electrode (LE410). The measured pH range was between 5.9 and 6.4 at 23 °C, 5.4 and 7.7 at 35 °C and 4.2 and 7.1 at 58 °C during the water storage time.

### 2.4. Melt Volume Rate

The melt volume rate (MVR) was measured on a mi2 (Göttfert Werkstoff-Prüfmaschinen GmbH, Buchen, Germany) according to ISO 1133. The temperature was set to 190 °C, and the stamp was loaded with a weight of 2.16 kg. Samples of 6 to 9 g, depending on the expected MVR, were weighed, and the heating time was set to 4 min. Prior to MVR measurements, the pellets were dried in a vacuum oven at 80 °C.

### 2.5. Aging of Phosphites

Samples were placed in a climate chamber WK11-180 (Weiss Technik GmbH, Reiskirchen-Lindenstruth, Germany). A relative humidity of 95% and a temperature of 58 °C were maintained for up to 1400 min.

### 2.6. Size-Exclusion Chromatography (SEC)

SEC measurements were performed using an SEC 1260 system (Agilent Technologies Deutschland GmbH, Waldbronn, Germany), consisting of a degasser (G1322A), isocratic pump (G1310B), autosampler (G1329B), thermostat (G1316A), refractive index detector (G7800A), two Agilent-PLgel-MIXED-C columns and a PLgel guard column. Chloroform was dried with a molecular sieve and used as eluent (c = 5 g L^−1^) at 35 °C with a flow rate of 1 mL min^−1^. Calibration was performed using polystyrene standards (PSS Polymer Standards Service GmbH, Mainz, Germany) over a molar mass distribution of 370–2,520,000 g mol^−1^. The injected sample volume was 100 µL. The standard deviations of SEC measurements were below 5% at the weight average molecular weight peak maximum.

### 2.7. Nuclear Magnetic Resonance (NMR)

Nuclear magnetic resonance (NMR) data were obtained on a Bruker NanoBay 300 spectrometer (7.05 T, Bruker, Ettlingen, Germany). The hydrolysis of phosphites aged in a climate chamber was analyzed with quantitative inverse-gated proton-decoupled ^31^P-NMR by solving a small amount of phosphite in 0.7 mL of CDCl_3_. ^31^P-NMR spectra were recorded at 27 °C, and for each spectrum, 16 scans with a relaxation delay of 15 s and a Lamour frequency of 121.60 MHz were applied. Chemical shifts are referenced to the lock signal of the solvent. Long-term hydrolysis of phosphites was analyzed with quantitative, inverse-gated proton-decoupled ^31^P-NMR under similar conditions, but with 24 scans in THF-d8 at 35 °C. A small amount of phosphite was diluted in 0.6 mL of THF-d8, and 0.1 mL of deionized water containing 1.8% (*v*/*v*) lactic acid was added. The sample was placed in the heated measuring chamber and measured immediately after preparation, every 30 min up to 16 h and after several more days, and the sample was then stored at 23 °C.

## 3. Results and Discussion

### 3.1. Processing Stability

Processing stability was quantified by melt volume rate measurements of unaged samples after extrusion on a twin-screw extruder. In order to test the thermal and thermo-oxidative degradation during the melt stage, different heating times were applied in MVR tests. Furthermore, size-exclusion experiments were carried out on selected samples.

**Figure 1:**
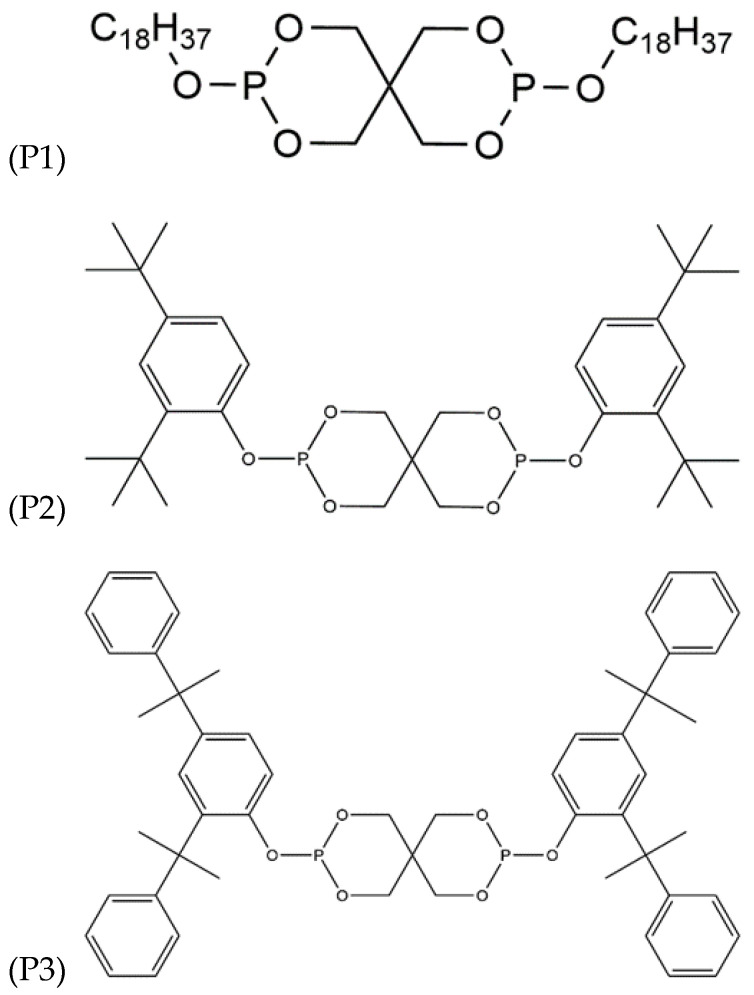
Chemical structures of phosphites: distearyl pentaerythritol diphosphate (P1), bis(2,4-di-tert-butylphenyl) pentaerythritol diphosphate (P2) and bis(2,4-dicumylphenyl) pentaerythritol diphosphate (P3).

PLA was compounded with three different substituted pentaerythritol-based diphosphites (chemical formula in Figure 1) with equimolar loadings. The MVR results shown in Table 1 indicate the severe degradation of PLA during extrusion when aliphatic phosphite P1 was added. A minor increase in MVR after extrusion was observed when P2 was added. With P3, there was no significant increase in MVR compared to the comparison sample after extrusion.

**Figure 2:**
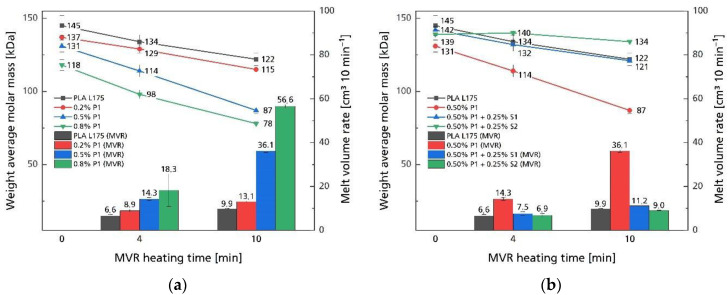
Melt volume rates and corresponding weight average molar mass depending on the MVR heating time of PLA samples with (**a**) different loadings of phosphite and (**b**) phosphite in combination with acid scavengers.

**Figure 3:**
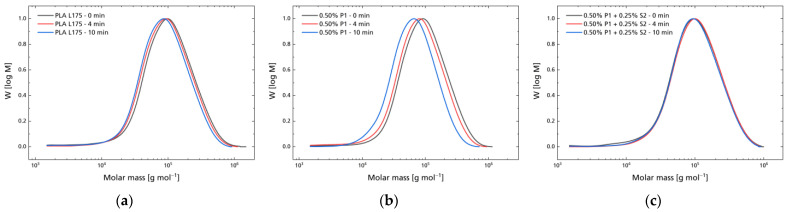
Molar mass distribution of samples after processing and after different MVR heating times at 190 °C: (**a**) PLA comparison sample; (**b**) 0.50% P1 added; (**c**) 0.50% P1 + 0.25% S2 added.

Striving to dive further into the influence on processing stability, different heating times during MVR measurements were applied, and molar mass distributions were assayed via SEC. Besides a heating time of 4 min, a longer heating time of 10 min was applied to evaluate the stability of the samples under typical processing temperatures for PLA. The obtained results clearly indicate that degradation during heating took place and depended on the loading of phosphite. With increasing concentration of P1, demonstrated in Figure 2a, there was a significant increase in MVR, i.e., a reduction in melt viscosity. At the same time, the reduction in weight average molar mass (${\overset{¯}{M}}_{w}$) reveals significant degradation during heating and is in accordance with MVR. With extended heating time, a stronger reduction in ${\overset{¯}{M}}_{w}$ and an increase in MVR were measured. Selected corresponding molar mass distributions are displayed in Figure 3a,b. They remained monomodal during heating, and a pronounced shift towards lower molar mass was observed when phosphite was added. These parameters clearly indicate a phosphite loading- and time-dependent reduction in the ${\overset{¯}{M}}_{w}$ of the investigated polyester in the melt state.

In contrast to the previously described chain extension of PLA in the melt state with triarylphosphites [59,65], the pentaerythritol-based phosphites examined did not improve the melt stability of PLA; in fact, they, more or less, reduced PLA melt stability under the conditions applied in this study, although this might be concentration-dependent. It was reported that a low concentration of TNPP increased the melt stability of PLA, but a higher concentration of TNPP resulted in an adverse effect, i.e., lower melt stability [59,65,66].

Jacques et al. [67] proposed several reaction mechanisms of triphenyl phosphites with terminal carboxyl and hydroxyl groups of PET/PBT blends leading to linear and branched chain extension with or without phosphorus in the polymer backbone.

**Scheme 1:**
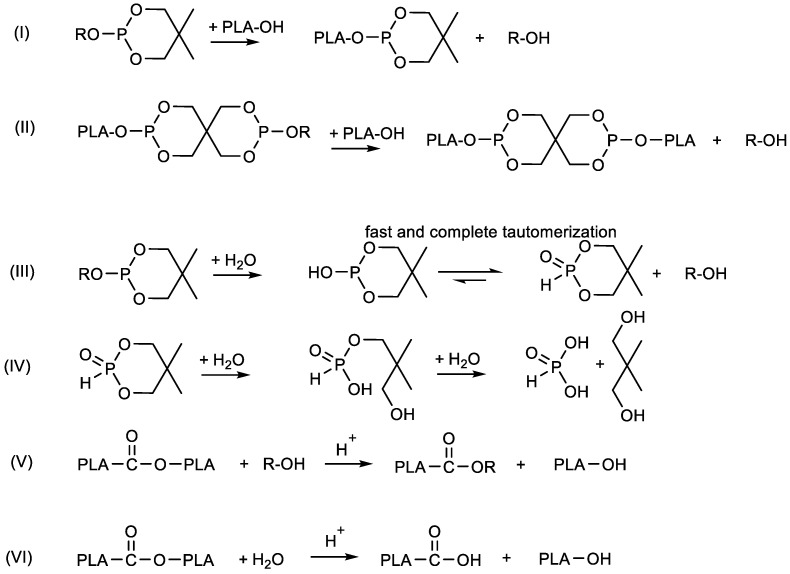
Proposed reactions of pentaerythritol phosphites in ring-opening-polymerized PLA in the presence of water. Reaction with terminal hydroxyl group of PLA (I); chain extension by reacting with a second terminal hydroxyl group (II); hydrolysis of P-O-R bond of phosphite (III); further hydrolysis of phosphite by cleaving of P-O-pentaerythritol bonds, releasing acidic products such as phosphorous acid (IV); acid-catalyzed transesterification of polymer ester with alcoholic degradation products released from phosphite (V); hydrolysis of PLA acid catalyzed by phosphorous acids and other acidic hydrolysis intermediates released from phosphite (VI).

Pentaerythritol-based phosphites contain two P-O-pentaerythritol and one P-O-R (R = alkyl or phenyl) bond, of which P-O-pentaerythritol bonds are more stable. Depending on the loading of phosphite, the concentration of terminal hydroxyl groups available in PLA and water concentration, we suggest the reactions proposed in Scheme 1. The kinetics depends heavily on the reactivity of the P-O-R bond determined by the alkyl or phenyl substituent.

Chain extension occurs preferentially when one pentaerythritol diphosphite reacts with two terminal hydroxyl groups of PLA, as shown in Scheme 1, reactions (I) and (II). When the majority of terminal hydroxyl groups react with a different phosphite moiety, there is no chain extension, presuming that reactions with P-O-pentaerythritol are negligible, until new reactive end groups are formed. When water is available, possibly supported by blocked hydroxyl end groups and high molecular weight resulting in a low concentration of terminal hydroxyl groups, then the hydrolysis of P-O-R with the formation of phosphonate (III) and, after that, the hydrolysis of P-O-pentaerythritol with the formation of phosphorous acid (IV) prevail. Finally, the acidic degradation products catalyze the transesterification of PLA backbone esters, with alcohols formed by the hydrolysis of phosphites or the end-chain reaction of phosphites with terminal hydroxyl groups (V), and catalyze the hydrolysis of esters in the PLA backbone when water is present (VI). These reactions likely occur simultaneously. The majority of the proposed reactions (excluding (II)) lead to a reduction in molecular weight or the release of degradation products catalyzing the molecular weight reduction. Since the molecular core and concentration of the examined phosphites are equal, i.e., pentaerythritol derivatives, the substituted R groups are decisive. It is known that the steric hindrance of P-O-R-bonds relates directly to the reactivity and hydrolytic stability of these phosphites [64,68]. Therefore, the high reactivity of alkyl-substituted phosphite P1 shows a negative impact on processing stability, while the highly aromatic-substituted phosphite P3 has no effect. The bis(2,4-di-tert-butylphenyl)-substituted phosphite P2 is in between P1 and P3 and, therefore, does not influence the processing stability to a high degree.

Golovy et al. [69] discovered that the hydrolysis of pentaerythritol phosphite and the subsequent formation of phosphonate are necessary for effective processing stabilization in PET and PBT blends. The phosphonate acts as a complexing agent with metal polymerization catalysts and inhibits transesterification reactions. Polycondensates are up to 0.2% metal residues, but in commercially produced PLA, tin (II) octoate is used for ring-opening polymerization at much lower concentrations [70]. As we observed an adverse effect here, this complexation with metal residues could also occur but is negligible under the applied experimental conditions.

A question is whether this possible shortcoming at the typical processing temperature can be inhibited by acid scavengers. Acid scavengers are used as stabilizers for phosphites to ensure their stability against hydrolysis [62]. When P1 is combined with acid scavengers, PLA is prevented from accelerated degradation (Figure 2b and Figure 3c). Both MVR and SEC verify that both acid scavengers (calcium stearate and hydrotalcite) act as stabilizers during the processing of PLA with phosphites. Moreover, the high acid capacity of hydrotalcites compared to calcium stearate [62] leads to a more stable system compared to neat PLA, even when phosphite is also added. When calcium stearate is combined with phosphite, the stability is close to that of the PLA comparison sample. Even very long heating times of 10 min do not lead to severe material degradation when hydrotalcite is used as a stabilizer. These results support the reactions suggested in Scheme 1. The acid scavengers prevent PLA as well as phosphite from hydrolysis, which would otherwise result in acidic and alcoholic degradation products. In the next step, these degradation products can autocatalytically decrease PLA molecular weight through catalyzing hydrolysis and transesterification, as explained above.

### 3.2. Hydrolytic Degradation

**Figure 4:**
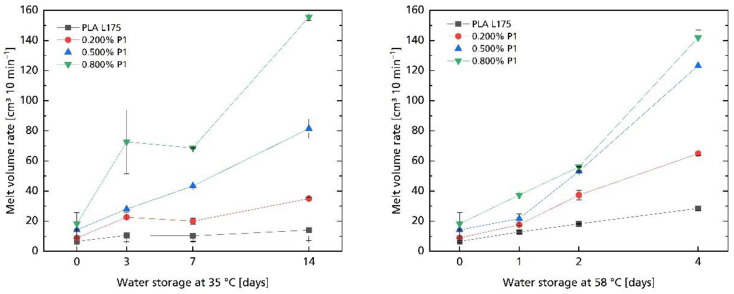

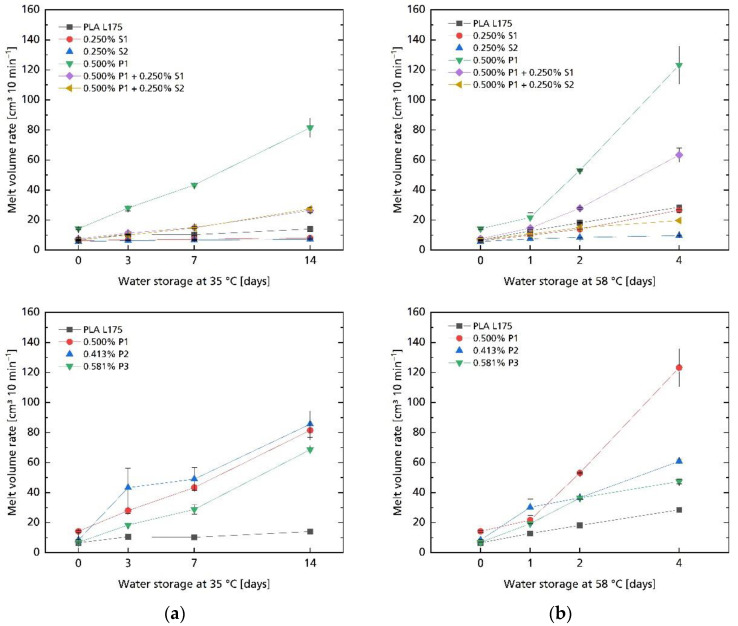
Melt volume rate of PLA pellets compounded with phosphites and acid scavengers during water storage at (**a**) 35 °C and (**b**) 58 °C.

With the aim to study whether the pentaerythritol phosphites under investigation enhance the hydrolysis of PLA and whether the used acid scavengers act as suitable agents to control such hydrolysis after ensuring processing stability beforehand, abiotic hydrolysis experiments were carried out. Abiotic degradation has a decisive role as a precursor to biotic degradation [12]. After extrusion, pellets were stored at 35 °C for up to 14 days and at 58 °C for up to 4 days in deionized water. In order to measure the viscosity, i.e., the molecular weight reduction during water storage, melt volume rate measurements were performed. The results are shown in Figure 4.

Neat PLA exhibited some degradation when stored in water at 35 °C since MVR was more than doubled after 14 days. At 58 °C, which is in the glass transition range of PLA, hydrolysis clearly accelerated. These results are in line with the previously described temperature dependency of PLA hydrolysis, particularly at and above the glass transition temperature [33]. The addition of phosphites resulted in an increase in the melt volume rate after the respective removal times. Looking at the performance of aliphatic phosphite P1, one can observe a faster increase in MVR compared to the PLA comparison sample. The amount of this increase depended on the added concentration of P1. In the next step, PLA compounded with 0.25% acid scavengers was tested. At 35 °C, both acid scavengers acted as equally effective stabilizers since the MVR increase was strongly reduced compared to the comparison sample. At 58 °C, only hydrotalcite still showed a pronounced effect, whereas the stabilization efficiency of calcium stearate was reduced and scarcely detectable after the fourth day. When 0.5% P1 was combined with 0.25% of either of the two acid scavengers, the hydrolytic stability was not affected in the first 3 days in relation to neat PLA at 35 °C, but hydrolysis was accelerated during the following water storage. With both acid scavengers, an equal quantitative increase in MVR was observable at 35 °C, whereas at 58 °C, the difference in the acid binding capacity [62] of the tested stabilizers led to different hydrolysis rates of the phosphite–stabilizer system too. When adding 0.25% hydrotalcite to 0.5% P1, the hydrolytic stability was still higher compared to neat PLA, even after 4 days at 58 °C. PLA with calcium stearate in combination with P1 was hydrolyzed only slightly faster than neat PLA during the first day at 58 °C in water storage, but the degradation clearly accelerated thereafter.

Turning to the performance of different equimolarly added phosphites (P1–P3), it can be stated that all types of phosphite accelerated polyester hydrolysis at both temperatures, but to varying extents. At 35 °C, the increase in MVR was less dependent on the phosphite type than at 58 °C. P2 was slightly more efficient than P1 at 35 °C, and at a higher temperature, P1 was the most efficient, followed by P2 and similarly P3. The hydrolytic stability of phosphites plays an important role. Through the hydrolysis of phosphites, finally, phosphoric and phosphorous acids will be released [62], and these are expected to act as catalysts for the acidic hydrolysis of PLA. Consequently, steric hindrance of P-O-R is expected to determine the kinetics of the proposed phosphorous acid-catalyzed hydrolysis of the polyester backbone. Beyond hydrolysis, other properties such as the solubility of phosphites in the polymer and migration and diffusion behavior might be contributing factors.

**Figure 5:**
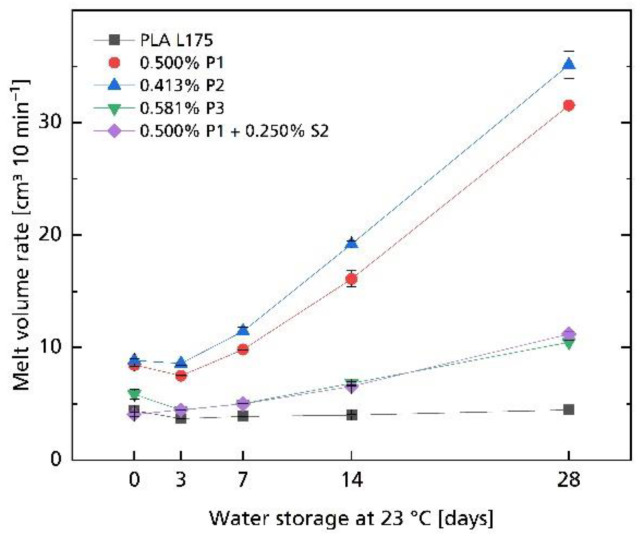
Melt volume rate of PLA pellets compounded with phosphites and acid scavengers during water storage at 23 °C.

Additionally, a second extrusion of PLA with the selected additives was performed, and samples were immersed in water at 23 °C for up to 28 days. Without phosphites, there was no increase in MVR for neat PLA at 23 °C, as can be seen in Figure 5. With P1 and P2 added, there was a strong increase in MVR after a lag time of several days, whereas the addition of P3 resulted in a less distinct increase in viscosity. P1 in combination with the high-acid-capacity stabilizer degraded to a lesser extent than P1 without a stabilizer, which proves the concept of accelerating and controlling the hydrolysis rate for ambient temperature as well.

SEC revealed the molar mass distributions and allowed the melt volume rate to be correlated with average molar mass. Table 2 shows the absolute and percentage retention of the number and weight average molar mass after storage in deionized water at 58 °C for 96 h. The results are in line with the corresponding measured MVR values. With increasing content of P1, a faster decrease in molar mass is observable, and acid scavengers in combination with phosphites at different intensities can delay the accelerated hydrolysis of PLA. All phosphites act catalytically on hydrolysis, where aliphatic-substituted phosphite P1 is the most effective under these conditions.

**Figure 6:**
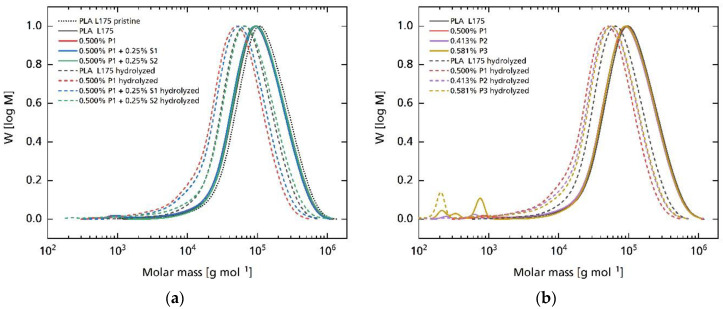
Molar mass distributions of PLA comparison sample and PLA with additives after processing and after 4 days of water storage at 58 °C: (**a**) P1 with acid scavengers; (**b**) different types of phosphites added in equimolar amounts.

In addition, normalized molar mass distributions are shown in Figure 6. The molar mass distributions remain monomodal in the observed timescale, indicating a homogeneous degradation in the bulk. This is probably due to the testing temperature being around glass transition, which facilitates fast and complete saturation with water molecules through the cross-section, and due to the non-buffered testing media, which minimizes the pH gradient towards the surface. There are some low molar mass signals detected when phosphites, especially the aromatic-substituted one, is added. These signals are apparent right after processing as well as after hydrolysis. Their existence can theoretically be explained by either mere additive signals or products from any interaction of the additive with the polymer. Their detected molar mass corresponds to the calculated molar mass of the additives, and they shift towards lower molar mass during water storage, which could be attributed to hydrolysis. Since they do not increase significantly, it is unlikely that these are related to lactic acid oligomers or other hydrolysis products of the polymer and therefore are not further considered for the calculation of average molar mass. There is a slight increase in the molar mass fraction of around 10 kDa. The latter can be explained by a predominantly autocatalytic and random-chain scission hydrolytic degradation pathway [33]. As expected, random-chain cleavage of the polyester backbone is evident. An almost immediate reduction in average molar mass is caused by random-chain cleavage [33]. However, it is very likely that end-chain scission also takes place because of a drop in pH during water storage, which is caused by the diffusion of low molecular weight compounds, such as lactic acid monomers and oligomers, into the hydrolysis medium. The molar mass distributions indicate that phosphites accelerate but do not change the degradation pattern.

**Figure 7:**
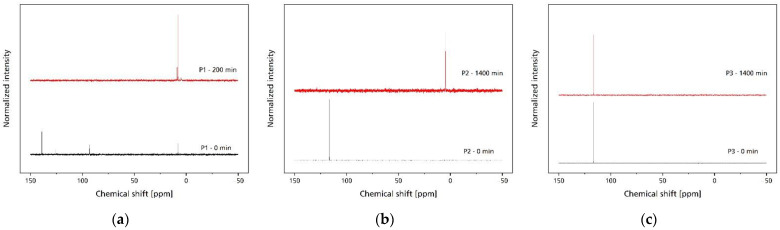
^31^P-NMR proton-decoupled spectra of different types of phosphites (**a**) P1, (**b**) P2 and (**c**) P3 in CDCl_3_ before and after exposure to 95% relative humidity and 58 °C.

To evaluate the hydrolytic stability of the phosphites used, they were aged in a climate chamber and examined with ^31^P-NMR. The results are presented in Figure 7. Phosphite P(OR)_3_ has a chemical shift δ_P_ of about 125–150 ppm. This value varies when bicyclic compounds such as spiro compounds are involved [71]. Distearyl pentaerythritol diphosphite, which contains only aliphatic groups, showed the complete degradation of P-O-R bonds after 200 min. In contrast, the highly aromatic-substituted bis(2,4-dicumylphenyl) pentaerythritol diphosphite showed no degradation after 1400 min. Bis(2,4-di-tert-butylphenyl) pentaerythritol diphosphate was fully hydrolyzed after 1400 min; however, there was no signal detected after 200 min. According to our theory that the hydrolysis of phosphites results in acidic degradation products, which hydrolyze PLA via acid catalysis, the hydrolytic instability of phosphites should correlate with the kinetics of accelerated degradation behavior. For the 58 °C testing temperature, this correlation is evident. The stearyl-substituted pentaerythritol diphosphite is most prone to hydrolysis under these conditions, and consequently, the sample containing P1 exhibits the fastest degradation. At temperatures below the glass transition of PLA, P2 seems to be as effective as P1, whereas P3 is clearly less effective in accelerating PLA hydrolysis. This is explained by the previously shown higher hydrolytic stability of P3, which slows down the hydrolysis of P3 and, in consequence, its ability to accelerate the degradation of PLA in the presence of water.

**Figure 8:**
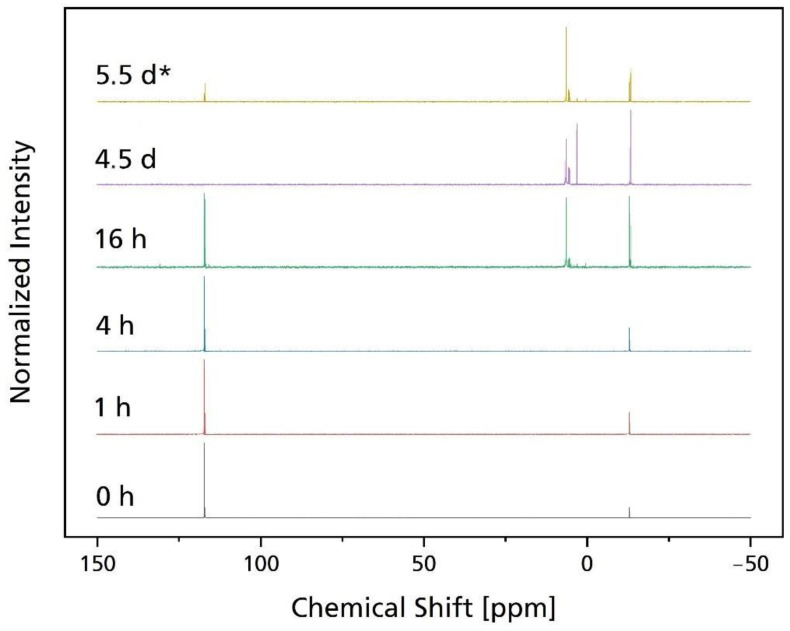
^31^P-NMR proton-decoupled spectra of bis(2,4-di-tert-butylphenyl) pentaerythritol diphosphite (P2) in 0.6 mL of THF-d8 with 0.1 mL of deionized water with 1.8% (*v*/*v*) lactic acid after different times; * without lactic acid.

The hydrolysis of bis(2,4-di-tert-butylphenyl) pentaerythritol diphosphite (P2) was further studied. P2 was diluted in THF, and water with lactic acid was added. The observed ^31^P-NMR proton-decoupled spectra of P2, plotted in Figure 8, show evidence of hydrolysis and oxidation of the phosphite.

**Figure 9:**
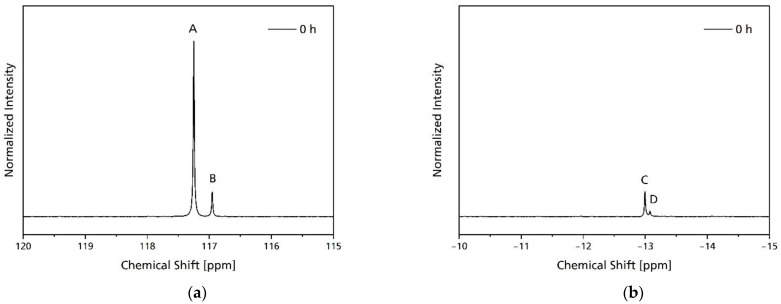
Initial ^31^P-NMR proton-decoupled spectra from 120 to 115 ppm (**a**) and −10 to −15 ppm (**b**) of bis(2,4-di-tert-butylphenyl) pentaerythritol diphosphite (P2) with evidence of pristine (A) and partly (B, C) or fully (D) oxidized phosphorous sites.

At the beginning and in the first hours, two signals, A (δ = 117.25 ppm) and B (δ = 116.95 ppm), are dominant (Figure 9a) and can be assigned to phosphite structures [56,71]. Additionally, two signals, C (δ = −12.99 ppm) and D (δ = −13.08 ppm), next to each other are visible (Figure 9b), which can be assigned to organic phosphate esters [56,71] formed through the oxidation of phosphite esters, which is initially most likely traced back to peroxides in THF leading to the rapid oxidation of the diluted sample, since these signals were not observed when Chloroform-d was used as the solvent.

**Figure 10:**
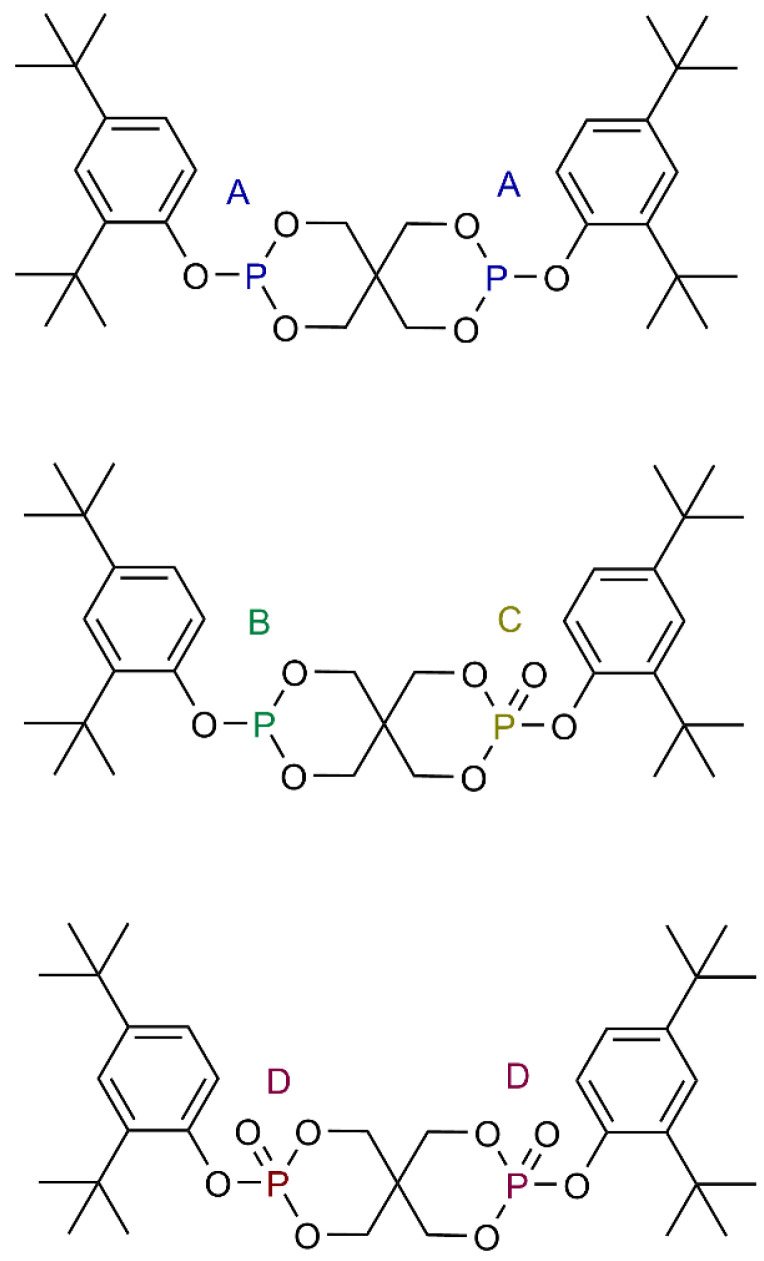
Proposed phosphite and phosphate structures attributed to ^31^P-NMR signals A, B, C and D.

Due to the symmetry of the molecule, only one pentaerythritol phosphite signal is expected. The integral of both signals B and C is very similar, and this suggests an equal quantity of phosphorous atoms related to B and C; i.e., they are associated. With respect to the expected oxidation products, the weaker phosphite signal B at 116.95 ppm can be attributed to one phosphate ester C present at the opposite site of pentaerythritol. Signal D only occurs when both phosphorous sites are in oxidized forms. The related structures are drawn in Figure 10.

With the progression of time, the hydrolysis and oxidation of phosphorous proceed. The phosphite resonance line intensities decrease due to hydrolysis, and the relation shifts towards an equal quantity of both phosphite signals A and B, indicating the oxidation of phosphorous. After 4 more days, neither signal is visible, and consequently, the phosphite degraded completely.

**Figure 11:**
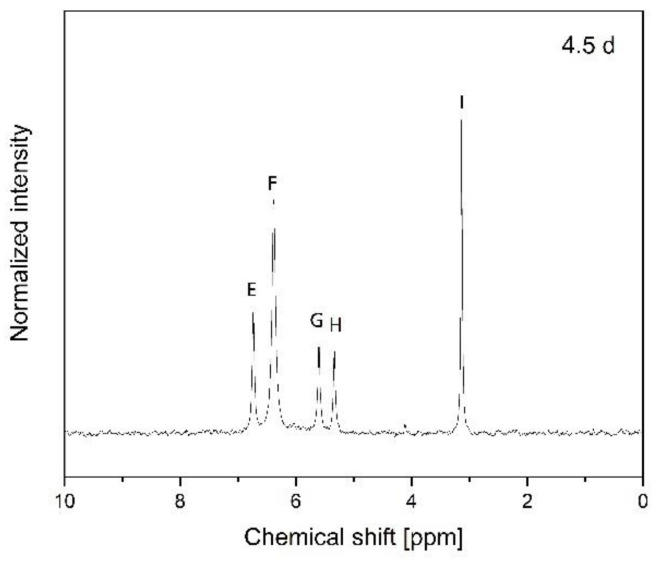
^31^P-NMR proton-decoupled spectra from 10 to 0 ppm of bis(2,4-di-tert-butylphenyl) pentaerythritol diphosphite (P2) in 0.6 mL of THF-d8 with 0.1 mL of deionized water with 1.8% (*v*/*v*) lactic acid after 4.5 days with signals corresponding to various hydrolysis products.

**Scheme 2:**
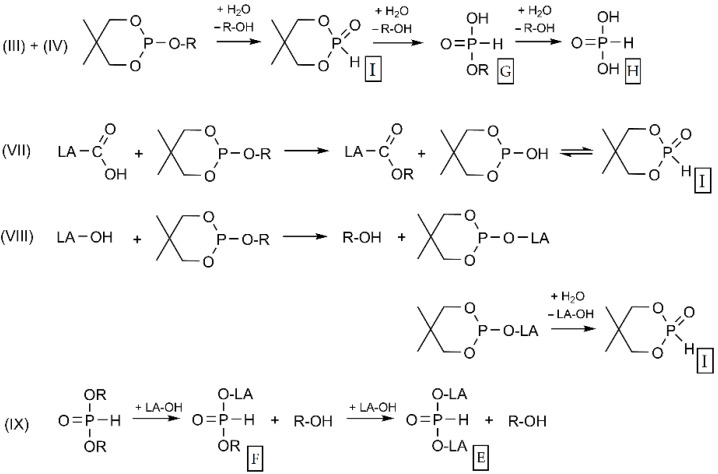
Proposed degradation products of pentaerythritol diphosphite associated with ^31^P-NMR signals E–I in the presence of lactic acid (LA).

Figure 11 shows the ^31^P-NMR spectrum after 4.5 days in the range from 0 to 10 ppm. Several ^31^P signals are present, which were not observed initially, but their formation started after about 8 h. These signals have been previously assigned to phosphite structures where a hydrogen atom is directly bonded to a phosphorous atom [56,71]. These bonds are in line with structures proposed as a result of the hydrolysis pathway of pentaerythritol diphosphites [56,62,72]. Consequently, signals E–I are attributed to hydrolysis products formed, according to the literature [56], and their proposed general structures are shown in Scheme 2. In the present study, lactic acid is present and can react through hydroxyl or carboxyl groups with the phosphite and its hydrolyzed and oxidized derivatives, which is also valid for PLA in real applications.

The addition of lactic acid accelerates the hydrolysis of phosphites, because in a similar experiment without lactic acid, there are no hydrolysis products (0–7 ppm) visible after 16 h. The hydrolyzed phosphite becomes detectable in the spectrum obtained after 5.5 days (Figure 8). There are still pentaerythritol phosphite signals detected after 5.5 days, which proves the slower hydrolysis kinetics when lactic acid is absent. The hydrolysis mechanism of phosphites proceeds autocatalytically [62], and that is why acid scavengers added in low concentrations to commercially available phosphites increase their hydrolytic stability [62].

## 4. Conclusions

An additive system consisting of pentaerythritol-based phosphite and an acid scavenger was identified as a practicable and effective solution for controlling the lifetime of biodegradable polyesters such as PLA. In this system, the phosphite acts as an accelerator for hydrolysis, and the acid scavenger functions as a stabilizer during processing and as a moderator of hydrolysis. The efficacy of the tested additives was evaluated by abiotic water storage at different temperatures and the subsequent determination of viscosity from the melt volume rate and molecular weight by SEC. Insights into the hydrolysis of phosphites when lactic acid is present were obtained through ^31^P-NMR. Based on the results, it is obvious that the abiotic hydrolysis of PLA can be controlled by modifying the loading and type of phosphite as well as by using acid scavengers, and these findings enable polymer design with a controlled degradation time. The presented additive system has the advantage of being active in the bulk and can be easily incorporated in PLA applications through standard processing methods, contrary to, e.g., enzymatic processes, where the activity is limited to the surface, and industrial processing into the polymer matrix is limited due to the high processing temperatures. Further studies are necessary to assess the accelerated biological degradation and to ensure that no significant negative effects on soil or biological activity in the environment are caused by the degradation products of the compounded polymer. However, it should be noted that aromatic degradation products such as phenol derivatives are generally not desired, and therefore, aliphatic-substituted phosphites are preferred from an environmental point of view. Future research targets include the extension of the proposed additive solution based on phosphites (and stabilizers) for improved biodegradability to other polyesters, such as PBS. However, for each polymer, targeted application and degradation environment, a tailor-made additive system should be designed to ensure optimal product properties and end of life.

## 5. Patents

Polidar, M.; Metzsch-Zilligen, E.; Pfaendner, R.; Hallstein, J. Use of hydroxycarboxylic acid salts for stabilizing thermoplastic condensation polymers, stabilized moulding compositions and moulding compostions produced therefrom WO2021214207 (A1). 28.10.2021.

Polidar, M.; Metzsch-Zilligen, E.; Pfaendner, R. Use of inorganic sulphites and/or thiosulphates for stabilising aliphatic polyesters, stabilised moulding compositions and moulding compositions and moulded parts produced therefrom WO2021213940 (A1). 28.10.2021.

Polidar, M.; Metzsch-Zilligen, E.; Pfaendner, R. Additive composition and use thereof, condensation polymer composition, molding compound and molding compounds produced therefrom, and molded parts and use thereof WO2021213939 (A1). 28.10.2021.

Polidar, M.; Metzsch-Zilligen, E.; Pfaendner, R. Additive composition and use thereof, condensation polymer composition, molding compound and molding compounds produced therefrom, and molded parts and use thereof WO2021213771 (A1). 28.10.2021.

Polidar, M.; Metzsch-Zilligen, E.; Pfaendner, R. Use of an additive composition for the controlled accelerated degradation of condensation polymers WO2020148089 (A1). 23.07.2020.

## Figures and Tables

**Table 1:** MVR after extrusion of comparison sample PLA L175 and with different types of phosphite added in equimolar amounts. Additionally, acid scavengers were used in combination with P1.

Compound	MVR in cm^3^ 10 min^−1^
PLA L175	6.62 ± 0.58
0.500% P1	14.28 ± 0.59
0.413% P2	8.40 ± 0.31
0.581% P3	6.89 ± 0.26
0.500% P1 + 0.25% S1	7.52 ± 0.86
0.500% P1 + 0.25% S2	6.87 ± 0.80

**Table 2:** Average molar mass by SEC after processing and retention after 4 days of hydrolysis at 58 °C of solid PLA samples, with retention in brackets.

Compound	${\overset{¯}{M}}_{n}$ * in kDa after Processing	${\overset{¯}{M}}_{n}$ * in kDa after Hydrolysis	${\overset{¯}{M}}_{w}$ * in kDa after Processing	${\overset{¯}{M}}_{w}$ * in kDa after Hydrolysis
PLA comparison	61.3	42.1 (68.7%)	139.0	91.4 (65.8%)
0.200% P1	56.6	31.5 (55.7%)	134.4	72.8 (54.2%)
0.500% P1	56.6	27.1 (47.9%)	134.6	65.0 (48.3%)
0.800% P1	56.8	24.0 (42.3%)	137.4	61.1 (44.5%)
0.413% P2	54.1	29.6 (54.7%)	132.2	73.5 (55.6%)
0.581% P3	62.4	31.6 (50.6%)	135.1	76.6 (56.7%)
0.500% P1 + 0.250% S1	55.4	30.1 (54.3%)	132.5	73.3 (55.3%)
0.500% P1 + 0.250% S2	66.3	38.4 (57.9%)	139.0	96.8 (69.6%)

* Calculated from >1.5 kDa.

## Data Availability

The data presented in this study are available on request from the corresponding author.
